# Does Adjuvant *Therapy* in Invasive Intraductal Papillary Mucinous Neoplasm of the Pancreas Improve Survival? A Systematic Review and Meta‐Analysis Using Trial Sequential Analysis

**DOI:** 10.1002/wjs.70187

**Published:** 2025-12-07

**Authors:** Carlo Ingaldi, Vincenzo D'Ambra, Claudio Ricci, Laura Alberici, Marina Migliori, Mariacristina Di Marco, Andrea Palloni, Cristina Mosconi, Carla Serra, Riccardo Casadei

**Affiliations:** ^1^ Division of Pancreatic Surgery IRCCS Azienda Ospedaliero‐Universitaria di Bologna Bologna Italy; ^2^ Department of Internal Medicine and Surgery (DIMEC), Alma Mater Studiorum University of Bologna S. Orsola‐Malpighi Hospital Bologna Italy; ^3^ Division of Internal Medicine IRCCS Azienda Ospedaliero‐Universitaria di Bologna Bologna Italy; ^4^ Division of Oncology IRCCS Azienda Ospedaliero‐Universitaria di Bologna Bologna Italy; ^5^ Division of Radiology IRCCS Azienda Ospedaliero‐Universitaria di Bologna Bologna Italy; ^6^ Division of Interventional Ultrasound IRCCS Azienda Ospedaliero‐Universitaria di Bologna Bologna Italy

**Keywords:** adjuvant therapy, IPMN, pancreatic cancer, survival

## Abstract

**Objective:**

This meta‐analysis aims to evaluate the efficacy of adjuvant *therapy* (ADJ) in patients with resected invasive IPMNs, compared with follow‐up (FUP).

**Methods:**

A random effects meta‐analysis was performed. Meta‐regression analysis was used to clarify heterogeneity. The trial sequential analysis was used to test Type I and Type II errors, defining the required information size (RIS). The primary endpoint was OS, and the secondary endpoint was DFS.

**Results:**

The accrued sample size (AIS) was 2422 patients for OS and 493 patients for DFS. OS and DFS in the ADJ arm were similar to the FUP arm (HR 1.21; 95% CI 0.81−1.79, *p* = 0.349 and HR 0.98; 95% CI 0.64−1.51, *p* = 0.936). The RIS were 2422 for OS and 254 for DFS, allowing the exclusion of Type II errors. For the primary endpoint, heterogeneity was high (I^2^ = 98%). Meta‐regression analysis showed that, although considering two groups equal for confounding covariates, OS in the ADJ and FUP arms remains similar. A subgroup analysis showed that node‐positive patients have improved OS after adjuvant *therapy* administration (HR 1.86; 95% CI 1.39; 2.47, *p* < 0.001).

**Conclusion:**

Adjuvant *therapy* should not be administered indiscriminately to all patients. Node‐positive invasive IPMN seems to have an improved OS.

**Trial Registration:**

PROSPERO 2024 CRD42024561326

## Graphical abstract

Question: What is the efficacy of adjuvant *therapy* in resected invasive IPMN? Findings: This meta‐analysis showed that patients with invasive IPMN do not benefit from adjuvant *therapy* (HR 1.21, 95% IC: 0.81−1.79). A subgroup analysis showed that node‐positive patients have an improved OS (HR 1.86; 95% CI 1.39; 2.47, *p* < 0.001). Meaning: Adjuvant *therapy* should not be administered indiscriminately to all patients. Node‐positive IPMN seems to have improved OS.

## 1 Introduction

Intraductal papillary mucinous neoplasms (IPMNs) are pancreatic cystic neoplasms originating from mucin‐producing epithelial cells lining the main pancreatic duct or its branch ducts. They are characterized by the secretion of thick mucin that can cause ductal dilation, forming visible cystic structures within the pancreas [1]. IPMNs carry a variable risk of progressing to invasive cancer, with higher risks observed in main duct (MD‐IPMNs) compared to branch duct (BD‐IPMNs) [2]. Approximately 20% of IPMNs may eventually develop into adenocarcinoma. Despite this risk, adenocarcinomas arising from IPMNs constitute only about 5% of all pancreatic adenocarcinomas, suggesting that other pathways dominate in most cases [3, 4].

Adjuvant therapy in pancreatic cancer is now well established. Recent trials, such as PRODIGE 24 and ESPAC‐4, have demonstrated improved overall survival with regimens such as FOLFIRINOX and gemcitabine plus capecitabine, respectively [5, 6]. These studies highlight the importance of adjuvant therapy in improving patient outcomes after surgery.

However, the survival benefit of adjuvant therapy in invasive IPMN remains unclear, and currently, there are no randomized trials addressing this issue [7].

The objective of this meta‐analysis is to evaluate the role of adjuvant therapy in patients with invasive cancer derived from IPMNs in terms of overall survival and disease‐free survival. Trial sequential analysis (TSA) methodology was employed to mitigate potential false positives or false negatives, due to small sample sizes [8, 9]. TSA systematically incorporates all published trials chronologically, estimating the required information size (RIS) necessary to conclusively accept or reject the null hypothesis while minimizing Type I and Type II errors [10, 11].

## 2 Methods

### 2.1 Search Strategy

A systematic review was carried out according to the Cochrane Handbook recommendations [12]. The search strategy was based on PICO's methodology [13]:

1. Population: patients with invasive intraductal papillary mucinous neoplasms (IPMN) who undergone surgery;
2. Intervention: patients undergoing adjuvant *therapy* (ADJ arm);
3. Control: patients undergoing follow‐up (FUP arm);
4. Outcomes: to evaluate overall survival (OS) and disease‐free survival (DFS).

A systematic literature search was performed using PubMed/Medline, Web of Science, and Scopus databases. Non‐English‐language studies were excluded. The search was conducted using the following string: “(‘IPMN’ OR ‘intraductal papillary mucinous neoplasm’ *OR* ‘*intraductal papillary mucinous carcinoma*’ OR ‘mucinous neoplasm’ OR ‘IPMNs’ OR ‘intraductal papillary mucinous neoplasms’ OR ‘mucinous neoplasms’) AND (‘malignant’ OR ‘invasive’ OR ‘adenocarcinoma’ OR ‘degenerated’ OR ‘degeneration’ OR ‘malignancy’ OR ‘carcinoma’ OR ‘cancer’) AND (‘adjuvant chemotherapy’ OR ‘chemotherapy’ OR ‘adjuvant’)”. The last research was performed on September 19, 2024. Studies were selected by reading titles and abstracts. In case of doubt, studies were selected by reading the full text to identify papers fulfilling the inclusion criteria. A PRISMA flowchart was built [14]. This protocol has been registered with PROSPERO 2024 CRD42024561326.

### 2.2 Inclusion, Exclusion Criteria, Data Collection Process, Items, and Risk of Bias Assessment

The inclusion criteria were (i) patients who underwent pancreatic resection, (ii) pathologically confirmed invasive IPMN, and (iii) studies reporting survival data.

Exclusion criteria were (i) unresected patients; (ii) PDAC histology (studies reporting patients with PDAC with concomitant IPMN were excluded); (iii) other organs dissemination or metastatic disease; (iv) non‐English‐language studies; (v) case reports, meta‐analyses, reviews, editorials, and expert opinions; and (vi) absence of a control group.

Studies with some exclusion criteria but with extractable data were included in the meta‐analysis. After a full‐text reading of selected studies, all relevant data and various shorts were collected in an Excel spreadsheet. Survival data were derived from the full‐text article. When the study did not report HR, it was obtained from Kaplan–Meier curve analysis according to the Parmar method [15, 16]. The following data were collected: author, year, country, study design, age, sex, type of operation (total pancreatectomy, pancreaticoduodenectomy, or left pancreatosplenectomy), TNM stage, perineural invasion (PNI), lymphvascular invasion (LVI), margin status (R0), grading, histology, OS, and DFS. The primary endpoint of this study was to evaluate OS among patients with invasive IPMN who underwent surgery and adjuvant chemoradiotherapy, compared with patients referred to surgery alone and follow‐up. The secondary endpoint was to evaluate DFS. The qualitative assessment of the studies was carried out using the validated methodological index for non‐randomized studies (MINORS) [17]. Two authors (VD and CI) extracted the data using a dedicated spreadsheet. Any disagreement was resolved after a collegial discussion involving the first authors.

### 2.3 Summary Measurements and Synthesis of the Results

All parameters were reported as frequencies with percentages or mean and standard deviation (SD). The Mantel–Haenszel random effects model was used to calculate the effect sizes [18]. The results were reported as hazard ratio (HR), risk ratio (RR) or mean difference (MD) with 95% confidence intervals (95 CI). A two‐tailed *p*‐value < 0.05 indicated a non‐negligible effect.

A TSA meta‐analysis was carried out to obtain two main measures: the measure of risk association, described as hazard ratio (RR), and the measure of credibility of the results, called required information size (RIS). RIS is the “a priori” sample size that should be reached to obtain results without incurring Type I and Type II errors [10]. RIS is obtained, considering the heterogeneity of included studies and setting the Type I error at 5% and the Type II error at 20% (power 80%) [11]. RIS was calculated for primary and secondary endpoints and reported in the Cartesian plane, in which the *y*‐axis indicates the Z‐score. At the same time, the *x*‐axis is the cumulative sample size. The Z‐score is a measure related to the *p*‐value; when it is higher than 1.96, the *p*‐value is less than 0.05. The Z‐curve can be obtained by adding each trial sequentially. The Z‐curve can cross two boundaries: the conventional (dotted green horizontal lines) and monitoring boundaries (dotted red lines). The conventional edge corresponds to the nominal *p*‐value = 0.05. False‐positive results (Type I error) are present when the Z‐curve crosses this limit, but RIS is not reached. The monitoring boundaries are the Z‐scores at which Type I errors can be excluded, and the results are credible. On the other hand, a false‐negative effect (Type II error) can be hypothesized when the Z‐curve remains within conventional and monitoring boundaries, and RIS is not yet reached. Finally, when the RIS is reached, with no significant effect, the Type II error can be rejected. In other words, any additional study is useless to show differences between the two arms [8, 9].

### 2.4 Risk of Bias Across Studies and Meta‐Regression Analysis

The risk of bias across included studies was tested by measuring both the “between‐study heterogeneity” and publication bias. The heterogeneity between studies was tested using *I* ^2^ [19]. The heterogeneity was interpreted as follows: If *I* ^2^ was  <  50%, the risk of “between‐study” heterogeneity was considered low to moderate, and if *I* ^2^ was  ≥  50%, it was judged high. The effect of confounding covariates was weighted with a meta‐regression analysis [20, 21]. In the first step, we calculated the distribution of confounding covariates among each arm, reporting the results as risk ratio (RR), mean difference (MD), or percentage. In the second step, the β coefficient with standard error (SE) and *R* ^2^ was reported. The beta coefficient ± SE was related to the change in the RR or MD of the event: A positive beta coefficient means that the covariate increase rate generates a positive HR modification. The *R* ^2^ measured the proportion, in percentage, of the heterogeneity explained by the variable. A two‐tailed *p*‐value < 0.05 was judged significant. The *p*‐values were also recalculated using Monte Carlo permutation to obtain solid results and to avoid overfitting. Finally, results were also presented as adjusted HR and 95% CI. Among the included papers, baseline data were found to be quite heterogeneous. For this reason, a random effect model was applied for the analysis. Publication bias was assessed with a funnel plot and Egger's and Begg's tests, and a *p*‐value <  0.05 indicated a non‐negligible “small‐study effect” [22, 23]. The statistical analysis was carried out using dedicated packages for R.

## 3 Results

### 3.1 Search Results and Baseline Characteristics

**FIGURE 1:**
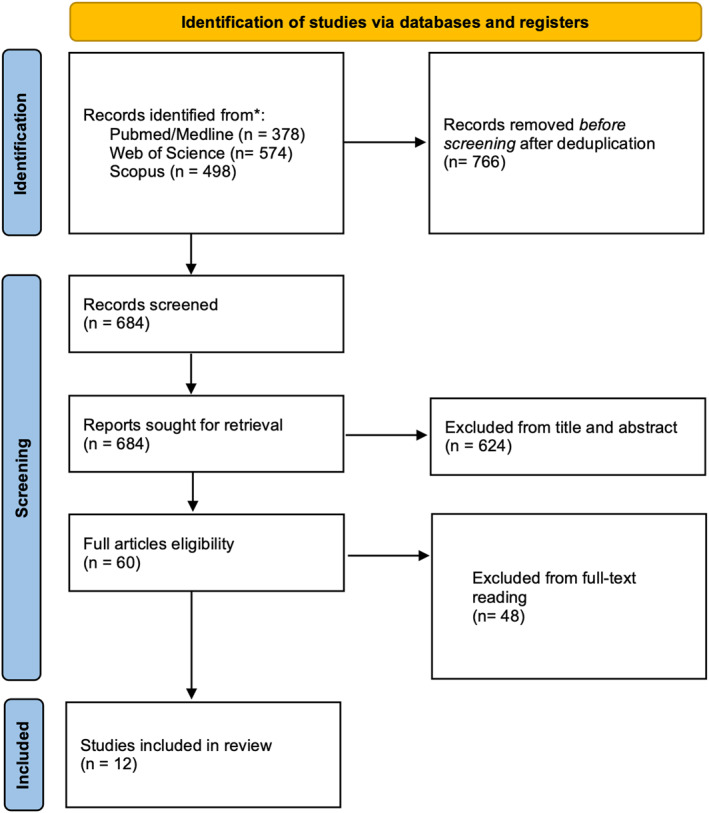
PRISMA flowchart of the study search and screening process.

The selection process is described in Figure 1. The search identified a total of *1450* records from Scopus, PubMed/Medline, and Web of Science; *766* studies were excluded after deduplication. Of the remaining *684* papers, *624* were excluded from the title and abstract because they were not pertinent to the field of the study. *Sixty* articles were reviewed, and 48 of these were excluded after full‐text reading. Finally, *12* studies were included in the analysis [24, 25, 26, 27, 28, 29, 30, 31, 32, 33, 34, 35]. The accrued sample size (AIS) was 2988 patients for the primary endpoint (OS): 1516 (45.7%) in arm adjuvant (ADJ) and 1472 (54.3%) in arm follow‐up (FUP). AIS was 493 patients for the secondary endpoint (DFS): 218 (44.2%) in arm adjuvant (ADJ) and 275 (55.8%) in arm follow‐up (FUP). Characteristics of the included studies are summarized in Table 1. Almost all studies (10%, *83.3%*) were conducted in Western countries and only 2 (*16.7%*) in Eastern countries. All were retrospective, and only *2* of them (16.7%) were conducted with PSM. The median value of the MINORS score was 16 [12, 13, 14, 15, 16, 17]. *Ten studies* reported data about overall survival, whereas only 4 reported data about disease‐free survival. The most popular chemotherapy regimens were 5‐fluorouracil or gemcitabine based, in some cases associated with radiotherapy.

**TABLE 1:** Characteristics of included studies (*n* = 12).

Author	Year	Country	Study design	MINORS	Sample size	Adjuvant	Control	Outcome evaluated	Adjuvant scheme based
Turrini et al.	2010	USA	Retrospective	16	98	37	61	OS, DFS	5‐FU, gemcitabine, radiotherapy
Swartz et al.	2010	USA	Retrospective	12	70	40	30	OS	5‐FU, radiotherapy
Caponi et al.	2013	Italy	Retrospective	16	64	33	31	OS, DFS	Gemcitabine, radiotherapy
McMillan et al.	2016	USA	Retrospective	16	1220	541	679	OS	Unknown
Duconseil et al.	2017	France	Retrospective	15	82	61	21	OS	Unknown
Marchegiani et al.	2018	Italy	Retrospective	16	44^a^	12^a^	32^a^	OS^a^	Gemcitabine, gemcitabine and oxaliplatin, 5‐FU and oxaliplatin, RT
Rodrigues et al.	2020	USA	Retrospective	16	103	34	69	OS, DFS	5‐FU, gemcitabine, radiotherapy
Choi et al.	2021	Korea	Retrospective	16	63	25	38	OS	Unknown
Habib et al.	2022	USA	Retrospective	14	213	93	120	OS	Unknown
Hirono et al.	2023	Japan	Retrospective with PSM	16	262^a^	147^a^	115^a^	OS^a^	S‐1, gemcitabine, gemcitabine, and S‐1
Lucocq et al.	2024	UK	Retrospective with PSM	17	228	114	114	OS, DFS	Gemcitabine, FOLFIRINOX, others unknown or radiotherapy
Habib et al.	2024	USA	Retrospective	16	847	538	309	OS	Gemcitabine, FOLFIRINOX, others (S‐1)

Abbreviations: 5‐FU, 5‐fluorouracil; DFS, disease‐free survival; MINORS, methodological index for non‐randomized studies; OS, overall survival; PSM, propensity score matching.

^a^ Extractable data were only for subgroup analysis of node‐positive patients.

### 3.2 Primary and Secondary Endpoints

**FIGURE 2:**
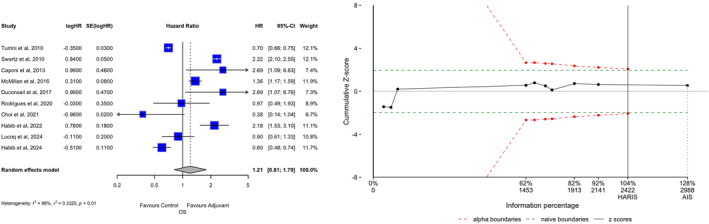
Overall survival (OS). (A) Forest plot. (B) TSA graph. The *x*‐axis is the number of patients; the *y*‐axis is the cumulative Z‐score value representing the effect of each arm; the continuous line is the cumulative Z‐score obtained by combining the studies; the dotted green horizontal lines are the conventional boundaries (*p*‐value < 0.05); when the Z‐curve crosses the conventional boundaries and the required information size (RIS) is not reached, the result is a false positive (Type I error); when the Z‐curve does not cross the conventional boundaries and RIS is not reached, the result is a false negative (Type II error); the dotted red logarithmic lines are the monitoring boundaries; when the Z‐curve crosses the monitoring boundaries, the result is a true positive. AIS, accrued sample size; CI, confidence interval; RIS, required information size; HR, hazard ratio; SE, standard error.

**FIGURE 3:**
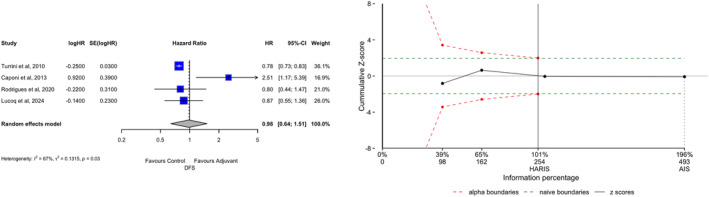
Disease‐free survival (DFS). (A) Forest plot. (B) TSA graph. The *x*‐axis is the number of patients; the *y*‐axis is the cumulative Z‐score value representing the effect of each arm; the continuous line is the cumulative Z‐score obtained by combining the studies; the dotted green horizontal lines are the conventional boundaries (*p*‐value < 0.05); when the Z‐curve crosses the conventional boundaries and the required information size (RIS) is not reached, the result is a false positive (Type I error); when the Z‐curve does not cross the conventional boundaries and RIS is not reached, the result is a false negative (Type II error); the dotted red logarithmic lines are the monitoring boundaries; when the Z‐curve crosses the monitoring boundaries, the result is a true positive. AIS, accrued sample size; CI, confidence interval; RIS, required information size; HR, hazard ratio; SE, standard error.

Results for primary and secondary endpoints are described in forest plots in Figures 2 and 3 and in Supporting Information S1: Table 1. Ten studies reported data about OS. OS in patients who underwent adjuvant chemoradiotherapy (ADJ) was similar to that of follow‐up (FUP) (HR 1.21; 95% CI 0.81−1.79, *p* = 0.165). The RIS of 2422 was reached, so Type II errors can be excluded (AIS = 2988, Δ = +566). Heterogeneity, however, was high (*I* ^2^ = 98%), whereas the small‐study effect was negligible (Egger *p* = 0.522, Begg *p* = 0.788, Supporting Information S1: Figure 1).

Four studies reported data about DFS. DFS in patients of the ADJ arm was similar to that of the FUP arm (HR 0.98; 95% CI 0.64−1.51, *p* = 0.936). The RIS of 254 was reached, so Type II errors can be excluded (AIS = 493, Δ = +239). Heterogeneity was high (*I* ^2^ = 67%), whereas the small‐study effect was negligible (Egger *p* = 0.312, Begg *p* = 0.174, Supporting Information S1: Figure 2).

### 3.3 Heterogeneity and Meta‐Regression Analysis

Meta‐regression analysis was conducted to explain the high heterogeneity for the primary endpoint (*I* ^2^ = 98%). Table 2 describes how the HR varies balancing two groups for confounding covariates. Considering two groups equal for confounding covariates, the adjusted HR did not vary substantially to become significant. Neither chemotherapy regimen significantly influenced the HR that means no regimen brought any survival advantage. As described in Supporting Information S1: Table 2 and in the bubble plot depicted in Supporting Information S1: Figure 3, low‐quality studies showed a clear survival advantage for patients undergoing adjuvant *therapy*, whereas high‐quality studies did not show significant differences in survival between the two groups (quality of studies explained a large degree of heterogeneity, *R* ^2^ = 47.9%).

**TABLE 2:** Univariate meta‐regression analysis for overall survival.

Confounding covariates	Effect on meta‐analytic measure^a^	
	HR	95% CI
Year of publication (2024) Propensity score matching (yes) MINORS (maximum) Western country Age (MD = 0) Male sex (RR = 1) PD (RR = 1) Stage I–II (RR = 1) T1‐2 stage (RR = 1) N0 (RR = 1) PNI (RR = 1) LVI (RR = 1) R0 (RR = 1) G3 (RR = 1) Tubular histology (RR = 1) 5‐FU (100%) Gemcitabine (100%) FOLFIRINOX (100%) RT (100%)	0.92 0.90 0.78 1.31 0.07 1.04 1.23 0.60 0.84 0.58 0.98 0.83 0.79 0.85 1.55 1.36 0.97 0.01 1.27	0.49–1.72 0.25–3.16 0.49–1.23 0.89–1.92 0.00–3.47 0.76–1.41 0.91–1.66 0.19–1.90 0.48–1.47 0.06–5.51 0.22–4.39 0.23–2.93 0.37–1.65 0.46–1.57 0.49–1.56 0.41–4.58 0.41–2.32 0.00–173.4 0.38–4.29

*Note:* The HR was adjusted for a fixed value of covariate, or fixed proportion (RR = 1) or fixed mean difference (MD = 0). In other words, this represents how the HR varies assuming the confounding covariate is equal in two groups or in the best scenario.

Abbreviations: 5‐FU, 5‐fluorouracil; CI, confidence interval; G3, high grade; HR, hazard ratio; LVI, lymphvascular invasion; MD, mean difference; MINORS, methodological index for non‐randomized studies; PD, pancreaticoduodenectomy; PNI, perineural invasion; PSM, propensity score matching; R0, radical resection with negative margins; RR, risk ratio; RT, radiotherapy.

^a^ Effect on the meta‐analytic result of the confounding covariate.

### 3.4 Subgroup Analysis of Node‐Positive Disease

Some of the included papers identified subcohorts of patients that may benefit from adjuvant *therapy*, such as node positive, high stage, R1 resection, and high grade. Unfortunately, only for the node‐positive subgroup, there were enough data to conduct a subanalysis.

**FIGURE 4:**
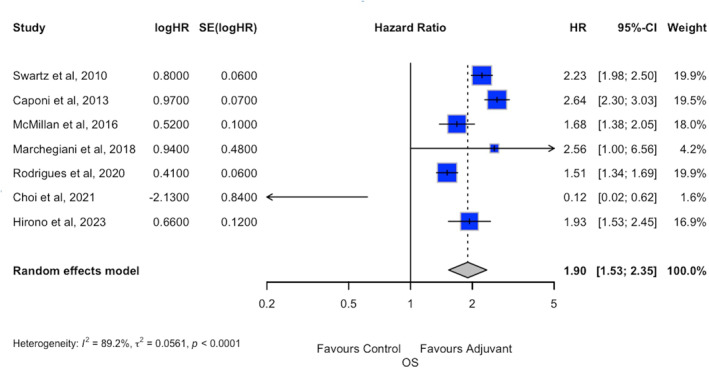
Subanalysis of node‐positive patients undergoing adjuvant chemotherapy compared with node‐positive patients undergoing follow‐up. Forest plot. CI, confidence interval; HR, hazard ratio; *I* ^2^, heterogeneity; OS, overall survival; SE, standard error.

The forest plot of overall survival is depicted in Figure 4. *Seven* studies reported data about this subgroup. Patients with node‐positive disease seem to benefit from adjuvant *therapy* administration (HR *1.90*; 95% CI *1.53*; *2.35*, *p < 0.001*).

Heterogeneity, however, was high (*I* ^*2*^ *= 89%*), whereas the small‐study effect was negligible (Egger *p* = *0.562*, Begg *p* = *0.761*).

## 4 Discussion

Adjuvant chemotherapy for PDAC is considered the standard of care, with randomized controlled trials demonstrating improved survival outcomes [5, 6]. On the contrary, the efficacy of adjuvant therapy for invasive IPMN remains controversial. The lack of clear guidelines on the administration of adjuvant chemotherapy is due to the absence of high‐quality evidence for invasive IPMN, combined with their generally less aggressive behavior and better prognosis [32]. This means that the decision to administer adjuvant chemotherapy is at the discretion of the oncologist and is usually based on histopathological characteristics, such as high grade, advanced stage, or lymph node positivity in the definitive histological examination. A previous systematic review by Aaronson et al. [7] concluded that adjuvant therapy may be beneficial in invasive IPMN in relation to specific tumor characteristics (node‐positive disease, positive resection margins, poorly differentiated tumors) with a tubular subtype. However, the nature of a simple review limited conclusive recommendations. The present meta‐analysis included *12* studies and assessed the OS and DFS of adjuvant therapy in invasive IPMN. This study is the first to include both OS and DFS, meta‐analysis, meta‐regression, and TSA analysis. This study suggests that in patients with invasive cancer derived from IPMNs, the administration of adjuvant chemoradiotherapy does not confer a clear advantage in terms of OS and DFS and should not be administered indiscriminately to all patients, regardless of the characteristics of the disease. The result is not affected by Type II errors owing to inadequate sample size, as highlighted in the TSA graph. On the other hand, the subgroup of patients with node‐positive disease seems to benefit from adjuvant chemotherapy administration.

This study has both strengths and limitations. The meta‐analytical results were validated using the TSA approach, which provides a measure of imprecision, specifically the classical *p*‐value, and validates the credibility of the results in estimating the presence of Type I or II errors. Additionally, TSA can assist pancreatic surgeons in evaluating the logic and utility of planning further randomized trials by calculating the correct sample size and appropriate endpoints [10, 11].

However, several limitations should be acknowledged. All included studies had a retrospective design, with only one study utilizing propensity score matching. This study exhibited high heterogeneity, and although explained by meta‐regression, its power may have been limited by missing data, potentially influencing survival outcomes. Some key data were lacking, and many data points were estimated using mathematical methods. Lastly, most included studies did not distinguish between different types of chemotherapy or different characteristics of treated patients.

We have to do some considerations. Firstly, the lack of subgroup analysis in many of the included studies, except for node‐positive patients, did not allow us to assess the role of chemotherapy in patients with different tumor characteristics (such as high‐grade, high‐stage disease, etc.). The survival benefit in patients with positive lymph nodes, as demonstrated by a recently published dataset [36] and by a recent systematic review [37], was confirmed by the aggregated data in our study. However, other tumor characteristics could benefit from its administration. Habib et al. [33] recently demonstrated, after multivariate and subgroup analysis, that patients with both node‐positive disease and elevated CA19‐9 have improved survival. Another study also showed improved survival in patients with a higher AJCC stage or a higher grade of disease [27]. We could speculate that patients with worse biological or anatomical tumor characteristics beyond node positivity could benefit from adjuvant chemotherapy, despite the result of this meta‐analysis. However, RCTs including a population with well‐defined tumor characteristics are needed.

Secondly, uncertainty of benefit in the administration of adjuvant chemotherapy in invasive IPMN still remains due to retrospective study design. Many retrospective studies, in fact, showed an advantage for adjuvant therapy, whereas many others did not. However, the meta‐regression allowed us to understand that this trend depends on the quality of the studies themselves. In fact, low‐quality studies tend to show a benefit from adjuvant therapy, whereas high‐quality studies do not. This confirms the need for high‐quality RCTs.

Thirdly, the trial sequential approach allowed us to understand that the required information size (RIS) has been reached for both endpoints. This means that there is no further scope for nonrandomized studies on this topic and confirms, once again, the need for RCTs to obtain more robust results and, if necessary, demonstrate some benefit of adjuvant chemoradiotherapy.

In conclusion, the present meta‐analysis suggests that adjuvant *therapy* should not be administered indiscriminately to all patients, regardless of the characteristics of the disease. Node‐positive invasive IPMN seems to benefit from its administration. However, given that these findings are based on low‐quality, retrospective studies and considering the limitations, as widely discussed, randomized controlled trials are needed.

## Author Contributions

**Carlo Ingaldi:** conceptualization, methodology, data curation, formal analysis, writing–original draft, writing–review and editing. **Vincenzo D'Ambra:** methodology, data curation, investigation, formal analysis, writing–original draft, writing–review and editing. **Claudio Ricci:** methodology, investigation, formal analysis, supervision. **Laura Alberici:** methodology, data curation, investigation, writing–original draft, writing–review and editing. **Marina Migliori:** conceptualization, methodology, supervision. **Mariacristina Di Marco:** conceptualization, methodology, supervision. **Andrea Palloni:** conceptualization, methodology, supervision. **Cristina Mosconi:** conceptualization, methodology, supervision. **Carla Serra:** conceptualization, methodology, supervision. **Riccardo Casadei:** conceptualization, methodology, supervision.

## Funding

The work reported in this publication was funded by the Italian Ministry of Health, RC‐2025‐2797188.

## Disclosures

This work was presented as a poster at the European Pancreatic Club (EPC) Annual Meeting, Düsseldorf, Germany, July 2–5, 2025.

## Conflicts of Interest

The authors declare no conflicts of interest.

## Supporting information


Supporting Information S1

## Data Availability

The data that support the findings of this study are available from the corresponding author upon reasonable request.
