# Supplementary material for: Does Adjuvant Therapy in Invasive Intraductal Papillary Mucinous Neoplasm of the Pancreas Improve Survival? A Systematic Review and Meta‐Analysis Using Trial Sequential Analysis
Source: World J Surg. 2025 Dec 7;50(1):196–203. doi: 10.1002/wjs.70187 (PMC12831527; doi:10.1002/wjs.70187)
Supplement: Supplementary file 1 — Supporting Information S1 [file WJS-50-196-s001.docx]

| Outcome of interest | No. | HR (95%CI) | P-value | I^2^ (%) | P-value for publication bias | | RIS | AIS | ∆ |
| --- | --- | --- | --- | --- | --- | --- | --- | --- | --- |
|  |  |  |  |  | Egger^ | Begg^ |  |  |  |
| *Primary Endpoint* |  |  |  |  |  |  |  |  |  |
| *OS* | 10 | 1.21 (0.81-1.79) | 0.349 | 98 | 0.522 | 0.788 | 2422 | 2988 | +566 |
| *Secondary Endpoint* |  |  |  |  |  |  |  |  |  |
| *DFS* | 4 | 0.98 (0.64-1.51) | 0.936 | 67 | 0.312 | 0.174 | 254 | 493 | +239 |

**Table 1.** Meta-analytic results.

**Legend:** HR= Hazard Ratio; I^2^= Higgins test for heterogeneity; ^Egger’s and Begg’s tests for publication bias; RIS= Required Information Size, RIS has been calculated considering a power of 0.80 and alpha = 0.05; AIS= Accrued Sample Size; ∆= AIS-RIS, a positive value means that RIS has been reached (no risk of type I or II errors); OS= Overall Survival; DFS= Disease Free Survival.


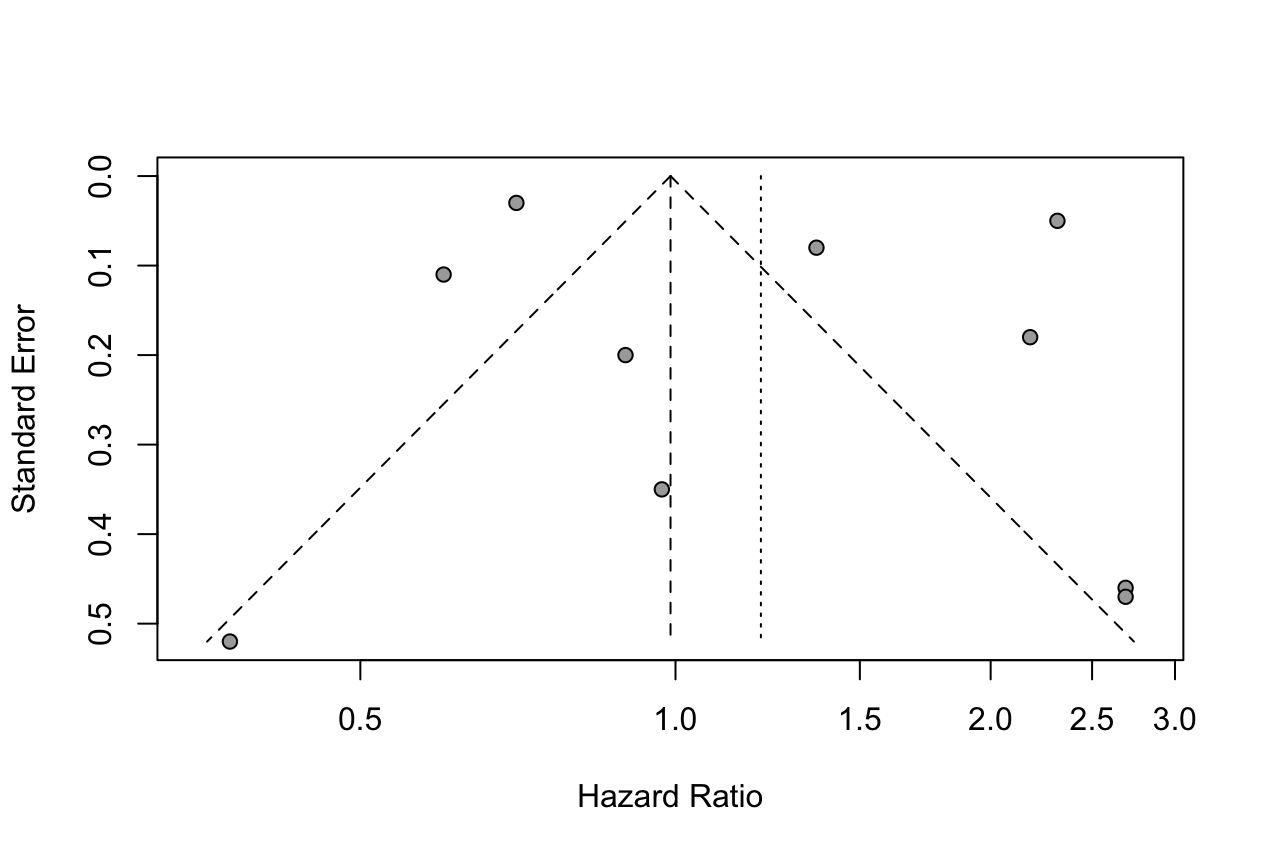


**Figure 1.** Funnel Plot for publication bias of OS.


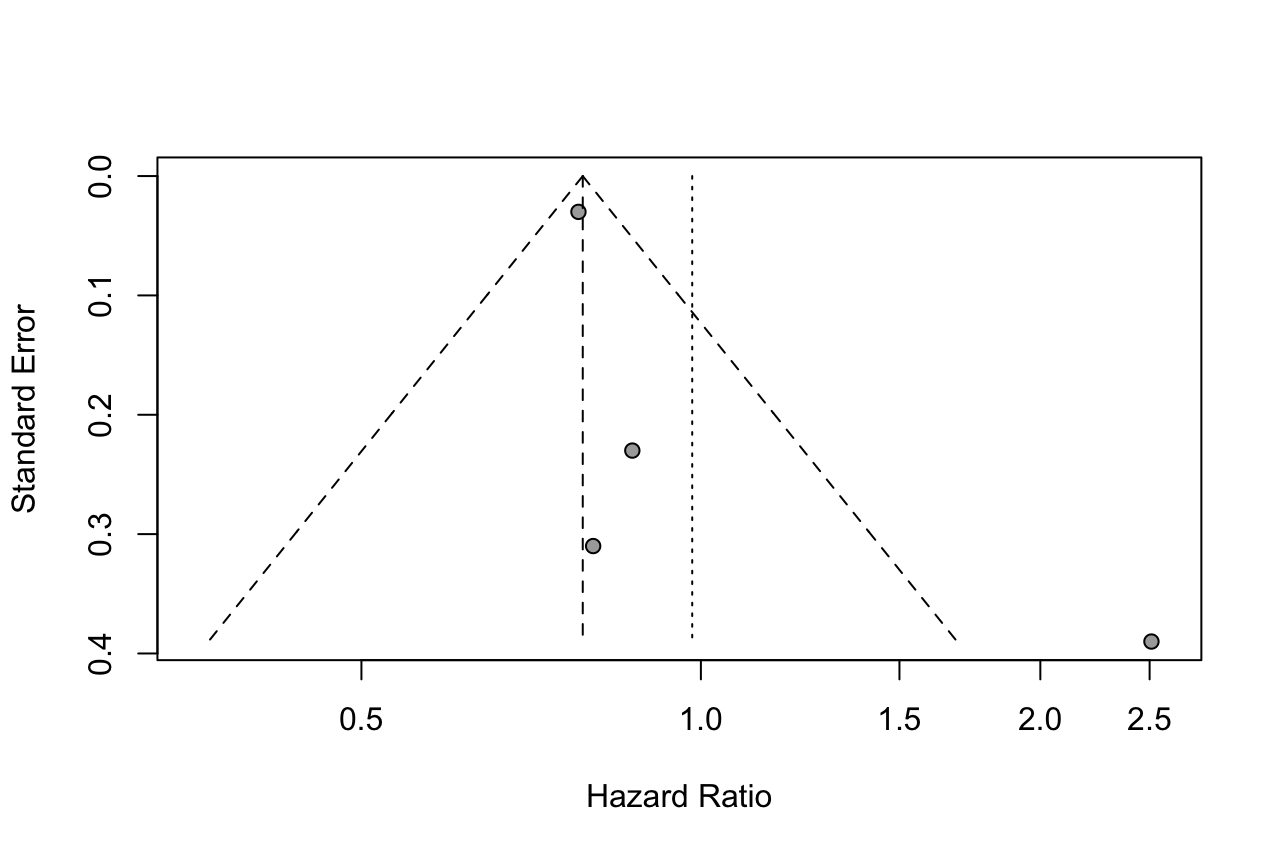


**Figure 2.** Funnel Plot for publication Bias of DFS.


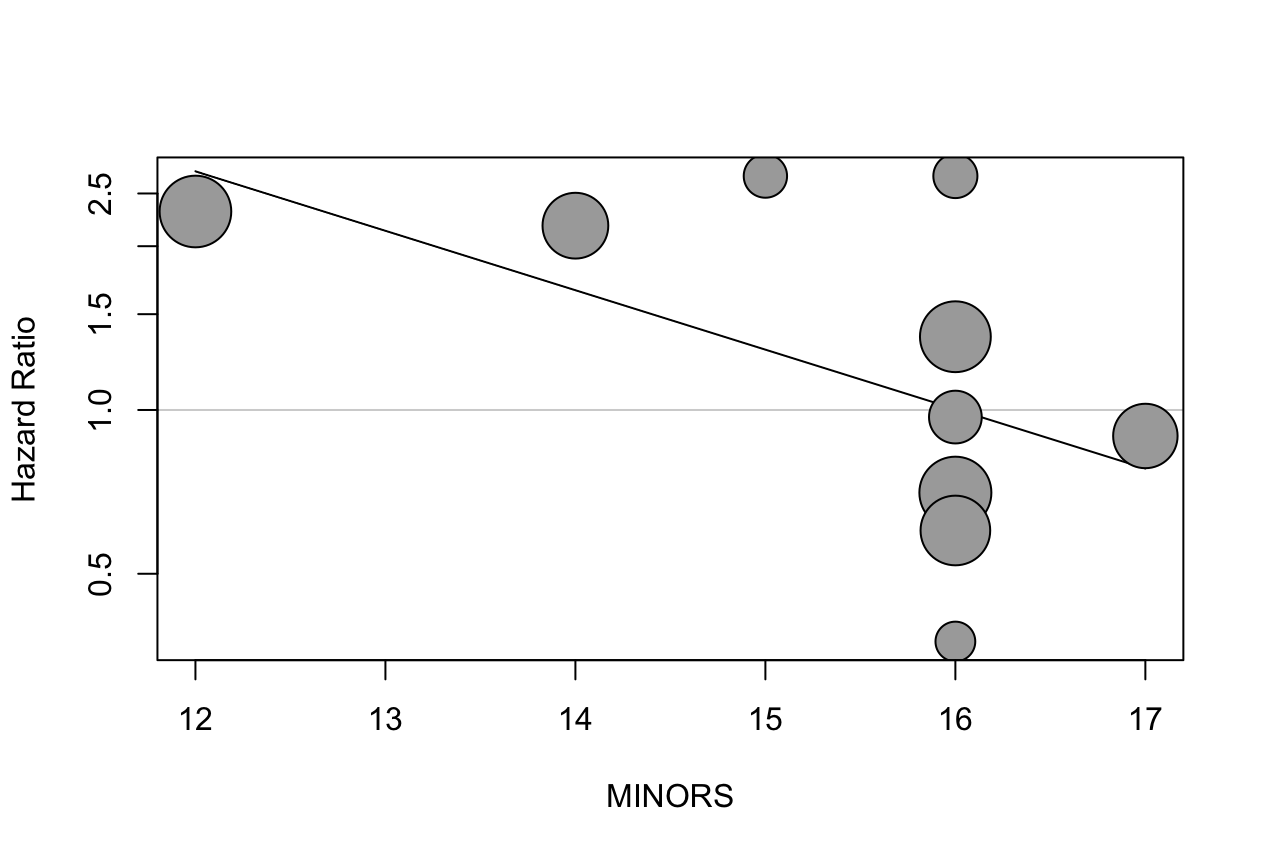


**Figure 3.** Bubble plot describing the relationship between quality of included studies (MINORS) and HR. To an increase of studies quality correspond to a decrease of HR (no differences of survival between two groups), and this can explain the 47.5% of heterogeneity (R2=47.5%, ß (SE)=-0.25 (0.10), p=0.014, after Monte Carlo Permutation p= 0.036).

| Covariates | Number of studies | Beta coefficient (SE) | Adjusted R^2^ (%) | P-value | P-value after Monte Carlo Permutation | Residue I^2^ (%) |
| --- | --- | --- | --- | --- | --- | --- |
| Year  PSM  MINORS  East-Western Study  Age MD  Male sex RR  PD RR  Stage I-II RR  T1-2 RR  N0 RR  PNI RR  LVI RR  R0 RR  G3 RR  Tubular Histology RR  5-FU (%)  Gemcitabine (%)  FOLFIRINOX (%)  RT (%) | 10  10  **10**  10  3  4  3  3  3  5  4  4  6  5  3  4  6  5  4 | -0.04 (0.04)  -0.33 (0.68)  **-0.25 (0.10)**  1.25 (0.78)  -0.52 (0.38)  5.86 (2.06)  7.03 (1.68)  -3.27 (3.96)  -1.15 (0.65)  -1.81 (2.34)  0.02 (0.71)  0.21 (0.52)  -3.23 (3.44)  1.25 (0.71)  0.23 (0.63)  -0.05 (0.94)  -0.26 (0.73)  -7.00 (6.24)  -0.31 (1.57) | 0.0  0.0  **47.9**  11.1  38.7  100  100  0.0  100  0.0  0.0  0.0  0.0  43.8  0.0  0.0  0.0  2.8  0.0 | 0.271  0.624  ***0.014***  0.109  0.164  0.005  <0.001  0.409  0.077  0.439  0.978  0.687  0.348  0.077  0.706  0.956  0.719  0.262  0.840 | 0.301  0.800  ***0.032***  0.100  0.500  0.083  0.167  0.667  0.167  0.517  1.000  0.833  0.417  0.175  1.000  0.875  0.678  0.300  0.916 | 97.5  97.9  **92.1**  97.5  67.3  0.0  0.0  67.1  0.0  98.3  87.8  92.7  97.6  94.5  71.7  98.6  98.3  98.8  97.6 |

**Table 2:** Univariate meta-regression analysis.

**Legend:** SE= standard error; R^2^ = relative reduction in between-study variance, the value indicates the proportion of between-study variance explained by covariate; Residue I^2^= Residue heterogeneity; MD= mean difference; RR= risk ratio;

**PSM = Propensity Score Matching; MINORS = Quality assessment using the Methodological Index for Non-Randomized Studies; PD= Pancreaticoduodenectomy; PNI= Perineural invasion; LVI= Lymph-vascular invasion; R0= radical resection with negative margins; G3= high grade; 5-FU= 5-Fluorouracil; RT= Radiotherapy.**
